# Antibacterial Effects of Cinnamon: From Farm to Food, Cosmetic and Pharmaceutical Industries

**DOI:** 10.3390/nu7095359

**Published:** 2015-09-11

**Authors:** Seyed Fazel Nabavi, Arianna Di Lorenzo, Morteza Izadi, Eduardo Sobarzo-Sánchez, Maria Daglia, Seyed Mohammad Nabavi

**Affiliations:** 1 Applied Biotechnology Research Center, Baqiyatallah University of Medical Sciences, P.O. Box 19395-5487, Tehran 14359-16471, Iran; E-Mail: Nabavisf@gmail.com; 2 Department of Drug Sciences, Medicinal Chemistry and Pharmaceutical Technology Section, University of Pavia, Pavia 27100, Italy; E-Mail: arianna.dilorenzo01@universitadipavia.it; 3 Health Research Center, Baqiyatallah University of Medical Sciences, Tehran 14359-16471, Iran; E-Mail: morteza_izadi@yahoo.com; 4 Laboratorio de Química Farmacéutica, Facultad de Farmacia, Universidad de Santiago de Compostela, Santiago de Compostela 15782, Spain; E-Mail: e.sobarzo@usc.es

**Keywords:** cinnamon, antibacterial activity, infectious diseases, spice, eugenol, cinnamaldehyde

## Abstract

Herbs and spices have been used since ancient times, because of their antimicrobial properties increasing the safety and shelf life of food products by acting against foodborne pathogens and spoilage bacteria. Plants have historically been used in traditional medicine as sources of natural antimicrobial substances for the treatment of infectious disease. Therefore, much attention has been paid to medicinal plants as a source of alternative antimicrobial strategies. Moreover, due to the growing demand for preservative-free cosmetics, herbal extracts with antimicrobial activity have recently been used in the cosmetic industry to reduce the risk of allergies connected to the presence of methylparabens. Some species belonging to the genus *Cinnamomum*, commonly used as spices, contain many antibacterial compounds. This paper reviews the literature published over the last five years regarding the antibacterial effects of cinnamon. In addition, a brief summary of the history, traditional uses, phytochemical constituents, and clinical impact of cinnamon is provided.

## 1. Introduction

Herbs and spices have been used since ancient times, not only as antioxidants and flavoring agents, but also for their antimicrobial activity against degradation induced by foodborne pathogens and food spoilage bacteria. Many plants used in traditional medicine represent rich sources of natural bioactive substances with health-promoting effects and no side effects. Nowadays, over 65% of the world population relies on traditional medicine for health care [1,2,3,4]. Recently, a large demand has risen for preservative-free cosmetics and antimicrobial herbal extracts, aimed at reducing the risk of allergies connected to synthetic preservatives such as methylparabens [5].

During the last two decades, growing evidence shows that plants are rich sources of different classes of antimicrobial substances acting as defense systems to protect them against biotic (living) and abiotic (non-living) stresses [6]. Among these secondary metabolites, polyphenols, terpenoids, alkaloids, lectins, polypeptides, and polyacetylenes are known to be antimicrobial agents; most of these metabolites are also approved as a GRAS (Generally Recognised as Safe) material for food products, showing negligible side effects. These properties give them special economic importance [7]. There are many edible and medicinal plants with high antimicrobial effects, such as thyme (*Thymus vulgaris* L.), tea (*Camellia sinensis* L.), garlic (*Allium sativum* L.), turmeric (*Curcuma longa* L.), berries belonging to Rosaceae family, and cinnamon (species belonging to *Cinnamomun* genus) [6,8].

The genus *Cinnamomum* (family Lauraceae) contains more than 300 evergreen aromatic trees and shrubs [9]. Four species have great economic importance for their multiple culinary uses as common spices worldwide: *Cinnamon zeylanicum* Blume (a synonym of *Cinnamon verum* J. Presl, known as Sri Lanka cinnamon), *Cinnamon loureiroi* Nees (known as Vietnamese cinnamon), *Cinnamon burmanni* (Nees & T. Nees) Blume (known as Indonesian cinnamon) and *Cinnamon aromaticum* Nees (a synonym of *Cinnamon cassia* (L.). J. Presl, known as Chinese cinnamon) [10]. The term cinnamon commonly refers to the dried bark of *C. zeylanicum* and *C. aromaticum* [11] used for the preparation of different types of chocolate, beverages, spicy candies and liquors [12]. Moreover, cinnamon is used in various savory dishes, pickles, soups, and Persian sweets. Cinnamon bark, leaves, flowers and fruits are used to prepare essential oils, which are destined for use in cosmetics or food products. Moreover, according to traditional Chinese medicine (dating roughly 4000 years), cinnamon has been used as a neuroprotective agent [13] and for the treatment of diabetes [14]. Cinnamon has also been used as a health-promoting agent for the treatment of diseases such as inflammation, gastrointestinal disorders and urinary infections [15,16]. Another potential medical use of cinnamon would be with regards to its antimicrobial properties, especially antibacterial activity. It is well known that infection is one of the leading causes of morbidity and mortality worldwide. According to the World Health Organization reports, in 2011, there were more than 55 million deaths worldwide with infection being responsible for one-third of all deaths [17]. The high prevalence of infection and long-term exposure to antibiotics has lead to the antibiotic resistance of microorganisms. Therefore, much attention has been paid to the discovery and development of new antimicrobial agents that might act against these resistant microorganisms, and cinnamon could be an interesting candidate [6,18].

The aim of this review is to analyze the available scientific data, published over the last five years, regarding the antibacterial effects of cinnamon and its active constituents such as cinnamaldehyde and eugenol. In addition, a brief summary on the history, cultivation, chemical composition, traditional uses, and clinical impacts of cinnamon is provided.

## 2. History

For thousands of years, cinnamon has been known as one of the most common spices, with multiple culinary usages [19]. In Ayurvedic medicine it has been used as antiemetic, anti-diarrheal, anti-flatulent, and stimulant agent [20]. Furthermore, it was used for embalming by the ancient Egyptian people [21]. In the 16th century, Portuguese conquistadors discovered *C. zeylanicum* growing widely in Sri Lanka, importing the spice to European countries during the 16th and 17th centuries [19]. During the Dutch occupation in the 17th century, cinnamon cultivation started in Java, and the East India Company became the main cinnamon exporter to European countries [21]. Although Ceylon cinnamon cultivation diminished, Sri Lanka remains the main source of cinnamon oils, and Ceylon cinnamon oil from Sri Lanka has been broadly used by both pharmaceutical and food industries. Pharmaceutical industries also use Chinese cinnamon oils [10,21].

## 3. Cultivation of Cinnamon

The average production rate of cinnamon is about 27,500 to 35,000 tons per year [22]. Cinnamon has mainly been cultivated in Sri Lanka, Seychelles, Madagascar and China [10,22,23]. In addition, it has been cultivated in India and Vietnam on a small scale [10,21]. Cinnamon can easily grow under tropical conditions in different soil types, ranging from the silver sands of the west coast of Sri Lanka to the loamy soils of its south coast. It has however been reported that soil quality and climate changes affect the production and quality of cinnamon. For example, the best cinnamon is produced in sandy soils enriched with humus. The optimum temperature for cinnamon cultivation is between 20–30 °C with an annual rainfall range of 1250–2500 mm. Cinnamon is commonly propagated by seedling and vegetative propagations. The application of fertilizer containing urea, phosphate, and potash is known to increase cinnamon production [23,24].

## 4. Chemical Composition of Cinnamon

**Figure 1:**
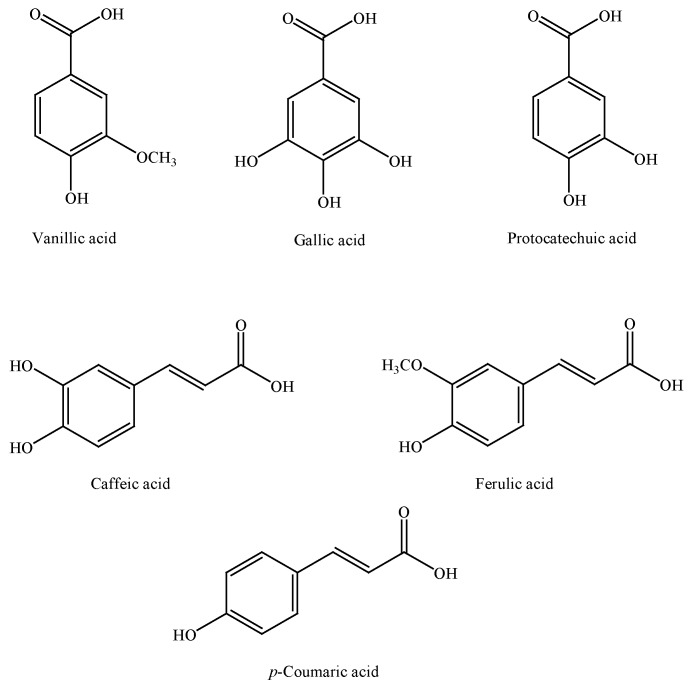
Polyphenolic constituents of cinnamon.

**Figure 2:**
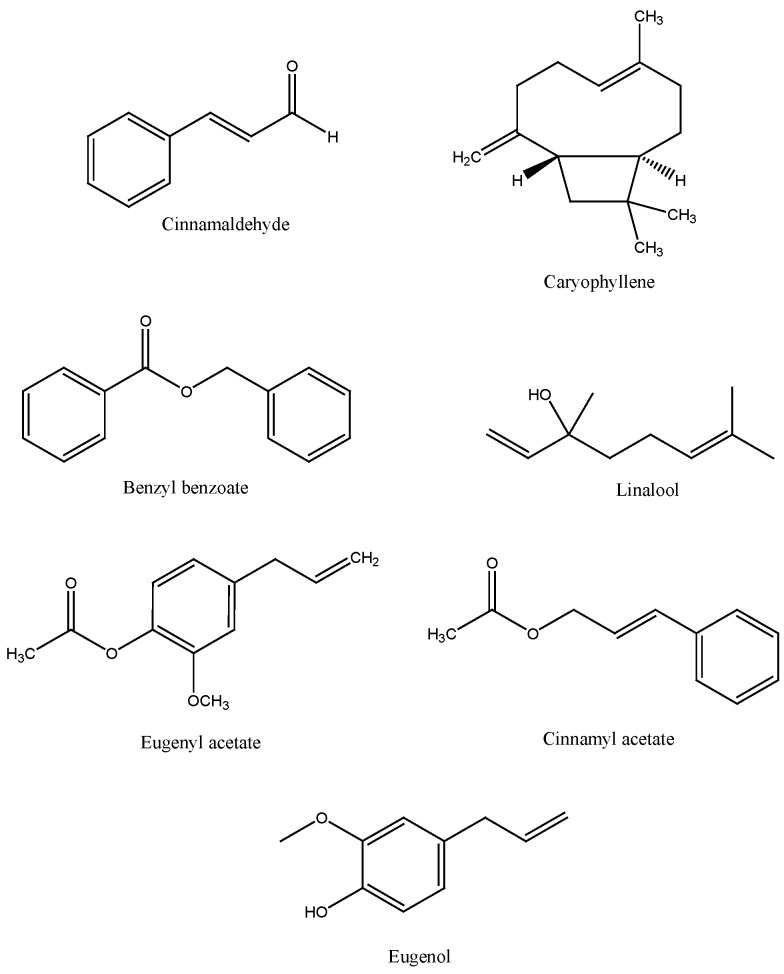
Major and minor chemical compounds of cinnamon essential oil.

The main compounds isolated and identified in cinnamon (*C. zeylanicum*) belong to two chemical classes: polyphenols and volatile phenols. Among polyphenols, cinnamon contains mainly vanillic, caffeic, gallic, protocatechuic, *p*-coumaric, and ferulic acids (Figure 1) [25]. With regards to volatile components, the chemical composition of cinnamon essential oils depends on the part of the plant from which they are extracted. In bark essential oil, cinnamaldehyde (Figure 2) is the most represented substance, with a content ranging from 90% to 62%–73%, depending on the type of extraction, this being higher for steam distillation than Soxhlet extraction [26]. The other minor volatile compounds are hydrocarbons and oxygenated compounds (*i.e.*, *β*-caryophyllene, benzyl benzoate, linalool, eugenyl acetate, and cinnamyl acetate) (Figure 2). In cinnamon leaf essential oil, the main component is eugenol, which reaches a concentration of more than 80%. In the essential oil obtained from cinnamon fruit and flowers, *(E)*-cinnamyl acetate and caryophyllene are the major components (Figure 2) [27,28,29].

## 5. Traditional Uses

Cinnamon has been known as one of the most common spices and food flavoring additives since ancient times [19]. For instance, it has been used as a flavor in sweets and chewing gum due to the pleasant and refreshing effect that develops in the mouth. It also shows beneficial effects on oral health and is used for toothaches, oral infections, and to remove bad breath [30]. Cinnamon has also been used to treat acne and melisma [31]. Moreover, it has been used for the treatment of gastrointestinal and colonic [32]. Ayurvedic literature shows that cinnamon has potent antiemetic, anti-diarrheal, anti-flatulent, and stimulant activities [33]. Cinnamon has a coagulant effect and therefore it can be used against hemorrhaging [34]. Cinnamon increases the blood flow in the uterus and improves tissue regeneration [35]. Moreover, it possesses potent antibacterial, antifungal, antitermitic, larvicidal, nematicidal, and insecticidal properties [14,36,37,38,39,40,41]. More recently, scientific reports showed that cinnamon has potent neuroprotective, hepatoprotective, cardioprotective and gastroprotective effects due to its potent antioxidant and anti-inflammatory properties [13,42]. Cinnamon essential oil could be also used in aromatherapy, which is the therapeutic use of plant essential oils that can be absorbed into the body via the skin or the olfactory system. A recent research articled showed the benefits deriving from the use of cinnamon oil in massage for alleviating menstrual pain [43].

## 6. Clinical Impacts

As far as the high therapeutic potential of cinnamon is concerned, there are numerous clinical studies on this spice. A search on the Clinical Trials Gov. database with the keyword “cinnamon” showed that there are 28 clinical trials, including 17 completed studies, six recruited studies, and one terminated study [44]. Most of these clinical trials are focused on its anti-diabetes and glucose lowering effects. In this context, some ongoing clinical trials are about the bioavailability of cinnamon and its beneficial effects on gingivitis, polycystic ovary syndrome, body fat, and blood glucose level in diabetic patients. Details of completed clinical trials on cinnamon are summarized in Table 1.

**Table 1:** Details of our search in http://clinicaltrial.gov website [44] with keyword “cinnamon”.

Clinical Trials	Title	Primary Outcome Measures and Treatments	Results
NCT02074423	A Human Clinical Trial Evaluating the Effect of MealShape™ on Blood Glucose Level Following Consumption of Standard Meal	measurements of blood glucose incremental area under the curve between 0 and 120 min, after consumption of a standard meal, compared the consumption of MealShape cinnamon extract (acute administration of 1 g corresponding to 2 capsules of 500 mg)	Cinnamon hydro-alcoholic extract may provide a natural and safe solution for the reduction of postprandial hyperglycemia and therefore help to reduce the risks of developing metabolic disorders.
NCT00846898	Is There a Metabolic Effect of Cinnamon on glycosylated hemoglobin A1c (HbA1c), Blood Pressure and Serum Lipids in Type 2 Diabetes Mellitus? (cinnamon)	measurements of blood profiles of HbA1c levels, after administration of cinnamon capsules (2 g per day for 12 weeks)	No study results posted on ClinicalTrials.gov* [44]
NCT00331279	The Effect of Cinnamon Extract on Insulin Resistance Parameters in Polycystic Ovary Syndrome: A Pilot Study	measurements of fasting glucose, fasting insulin, Homeostasis Model Assessment – Insulin Resistance (HOMA-IR), Quantitative Insulin Sensitivity Check Index (QUICKI), insulin sensitivity index (Matsuda), after administration of 2 cinnamon tablets (500 mg of purified aqueous extract of cinnamon for 8 weeks).	No study results posted on ClinicalTrials.gov* [44]
NCT00951639	Cassia Cinnamon for Glucose Uptake In Young Women	measurements of blood glucose, after the treatment with a cinnamon food supplement (5 g encapsulated ground bark administered once in experimental session)	No study results posted on ClinicalTrials.gov* [44]
NCT00237640	Effect of Cinnamon on Glucose and Lipid Levels in Non-Insulin Dependent Type 2 Diabetes Mellitus	measurements of HbA1c, glucose, total cholesterol, low-density lipoprotein (LDL cholesterol), high-density lipoprotein (HDL cholesterol), and triglycerides levels, after the treatment with cinnamon (500 mg capsule twice daily for 3 months)	Cinnamon taken at a dose of 1 g daily for 3 months produced no significant change in fasting glucose, lipid, A1C, or insulin levels.
NCT00371800	The Effect of Cinnamon on HbA1c Among Adolescents With Type I Diabetes	measurements of blood profiles of HbA1c levels, after the treatment with cinnamon (1 gram/day for 90 days).	No study results posted on ClinicalTrials.gov* [44]
NCT01350284	The Effect of Natural Food Flavourings on Gastrointestinal and Cardiovascular Physiological Responses. (CinnGastEmpt)	measurements of the effect of 3 g cinnamon on gastric emptying half time	An aliquot of 3 g cinnamon did not alter the postprandial response to a high-fat test meal. No evidence was found to support the use of 3 g cinnamon supplementation for the prevention or treatment of metabolic disease
NCT01027585	The Effects of Cinnamon on Postprandial Blood Glucose, and Insulin in Subjects With Impaired Glucose Tolerance	measurements of postprandial blood glucose, and plasma concentrations of insulin in subjects with impaired glucose tolerance, after the treatment with cinnamon capsules (doses not provided, for 5 months)	No study results posted on ClinicalTrials.gov* [44]
NCT01085019	Impact of Spices and Herbs on Endothelial Function	measurements of circulating level of plasma lipoproteins-lipids, oxidative stress, endothelial activation and inflammatory markers, after daily consumption of spices and herbs, among which cinnamon in capsules (2.8 g/day for 4 weeks)	No study results posted on ClinicalTrials.gov* [44]
NCT00718796	Naturopathic Treatment for the Prevention of Cardiovascular Disease (CVD)	evaluation of metabolic syndrome and general cardiovascular risk profile (Framingham Heart Study), after naturopathic approach with some spices (among which cinnamon) to CVD prevention over the course of 1 year	Naturopathic approach to CVD primary prevention significantly reduced CVD risk over usual care plus biometric screening and reduced costs to society and employers in this multi-worksite-based study.
NCT02193438	Physiologic Effect of Spices Ingestion	determination of resting energy expenditure, calculation of the resting energy expenditure from continuous measurement of oxygen consumption and carbon dioxid production (indirect calorimetry), heart rate variability, power spectral analysis of heart rate variability from continuous measurement of very low, low and high frequency range electrocardiographic signals, after the ingestion of a single dose of cinnamon extract (dose not provided).	No study results posted on ClinicalTrials.gov* [44]
NCT02234206	A Clinical Trial to Study the Safety and Efficacy of Chandrakanthi Choornam in Patients With Low Sperm Count	measurements of sperm concentration, proportion of sperm motility changes in the percentage of total and progressive motility of sperm proportion of sperm morphology changes in the percentage of sperm cells with normal forms, after the treatment with Chandrakanthi Choornam (dose non provided), which is a formulation consisting of 25 ingredients, among which Cinnamomum verum (bark) and Cinnamomum tamala (leaf) for 3 months	No study results posted on ClinicalTrials.gov* [44]
NCT00954902	Effects of Antioxidants on Cardiovascular Risk Measures (Spice Study)	measurements of Interleukin 6 (IL-6) response to psychological stress at time points equal to and greater than 90 min post task, after treatment with a high antioxidant spice blend (14.5 g blend of spice, among which cinnamon, incorporated into a delivery meal	Inclusion of spices may attenuate postprandial lipemia via inhibition of Phospholipase (PL) and Phospholipase A_2_ (PLA_2_).
NCT01752868	Can Fish Oil and Phytochemical Supplements Mimic Anti-Aging Effects of Calorie Restriction?	measurements of carotid-femoral pulse wave velocity, after the treatment with a combination of 10 nutritional supplements, among which cinnamon bark, for 6 months	No study results posted on ClinicalTrials.gov* [44]
NCT01667523	The Effect of Capsaicin and Cinnamaldehyde on Intestinal Permeability	evaluation of the effect of capsaicin and cinnamaldehyde infusion on intestinal permeability, after the administration of cinnamaldehyde (70 mg per intervention administered intraduodenally)	No study results posted on ClinicalTrials.gov* [44]
NCT01895816	Herbal Tonic Fertile Supplement(ZO2C5)	measurements of sperm count variation and semen analysis according World Health Organization methods, after the treatment with mixed herbals drug, in which cinnamon is one of the bioactive components for 6 months (dose not provided)	No study results posted on ClinicalTrials.gov* [44]

* as reported in http://clinicaltrial.gov/ website, the absence of posted results could be due to the facts that: the study may not be subject to U.S. Federal requirements to submit results, or the study has been completed, but the deadline for results submission has not passed, or the results have been submitted but have not yet been posted (for example, pending review by ClinicalTrials.gov) [44].

## 7. Antibacterial Effects of Cinnamon Essential Oil and Cinnamon Extracts

One of the most well-established properties of cinnamon extracts, essential oils and their components is the antibacterial activity against Gram-positive and Gram-negative bacteria responsible for human infectious diseases and degradation of food or cosmetics. In the literature, there are a number of studies showing the antibacterial activity of cinnamon essential oils obtained from different botanical parts and extraction methods. A search was conducted on the PubMed database [45], using the keywords “*antibacterial activity of cinnamon*”. The results returned 45 papers from 2010 up to 2015; the most interesting of these were summarized and critically discussed to provide a consistent review.

### 7.1. Antibacterial Activity of Cinnamon against Bacteria Responsible for Human Infectious Diseases

In 2011, the antibacterial activities of several *C. zeylanicum* bark extracts, obtained with different organic solvents, as ethyl acetate, acetone and methanol, were tested *in vitro* against *Klebsiella pneumonia* 13883, *Bacillus megaterium* NRS, *Pseudomonas aeruginosa* ATCC 27859, *Staphylococcus aureus* 6538 P, *Escherichia coli* ATCC 8739, *Enterobacter cloacae* ATCC 13047, *Corynebacterium xerosis* UC 9165, *Streptococcus faecalis* DC 74, by the disk-diffusion method. The results showed that the antibacterial activity, expressed as inhibition zone, ranges from 7 to 18 mm for the application of 30 µL, suggesting a high antibacterial activity [46]. In the same year, Mandal *et al.* showed that the ethanolic extract of stem bark *C. zeylanicum* exerted antibacterial activity against clinical isolates of methicillin resistant *S. aureus* (MRSA), from Kolkata, India. The antibacterial activity was expressed as both diameters of inhibition and minimum inhibitory concentration (MIC) values at different times of incubation. The cinnamon extract, which showed a diameter of inhibition zone ranging from 22 to 27 mm, resulted to be bactericidal after 6 h of incubation. The authors concluded that *C. zeylanicum* could be considered a valuable support in the treatment of infection and may contribute to the development of potential antimicrobial agents against MRSA bacteria [47].

As part of the studies on the antibacterial activity of cinnamon, the sensibility of two clinical strains of *Moraxella catarrhalis* (an important cause of lower respiratory tract infection, resistant to conventional antimicrobial agents) to the hydro-ethanolic extract of *C. zeylanicum* bark and clove powder, was tested using disk-diffusion and broth dilution methods. The results showed that cinnamon extract is active against both strains and, therefore, it represents an alternative source of natural antimicrobial substances for use in clinical practice for the treatment of cases of *M. cattarhalis* [48]. In 2012, Guerra *et al.* published an investigation on the antibacterial activity of the combination of *C. zeylanicum* essential oil and antibiotics, in which additive and synergistic effects were shown [49]. More recently, Yap *et al.* reached similar results. In fact, the authors showed that the combination of piperacillin and cinnamon bark essential oil induced a considerable reduction in the registered MIC values against a clinical strain of beta-lactamase-producing *E. coli*. The authors concluded that a reduced use of antibiotics could be employed as a treatment strategy to decrease the adverse effects and possibly to reverse the beta-lactam antibiotic resistance [50].

In the same year, cinnamon bark essential oil obtained through hydro-distillation was tested for antibacterial activity (expressed as MIC) against several pathogenic bacterial strains (*Salmonella typhi*, *Salmonella paratyphi A*, *E. coli*, *S. aureus*, *Pseudomonas fluorescens* and *Bacillus licheniformis*) and analyzed with thin layer chromatography (TLC) and gas chromatography coupled with mass spectrometry (GC-MS). The results showed that the tested sample exhibited excellent activity against all the selected strains (MIC values ranged from 2.9 to 4.8 mg/mL). TLC and GC-MS analyses of chemical composition revealed the presence of *t*-cinnamaldehyde (which was the most abundant substance, corresponding to 4.3%), eugenol (0.32%) and minor components such as cuminaldehyde, and γ-terpinene [51].

In 2014, Al-Mariri and Safi studied the antibacterial activity against Gram-negative bacteria (using a microdilution broth susceptibility assay) of cinnamon bark essential oil obtained via hydro-steam distillation. The sample showed good antibacterial activity against the Gram-negative bacteria (*E. coli* O157:H7, *Yersinia enterocolitica* O9, *Proteus* spp. and *Klebsiella pneumonia*) with very low MIC values (12.5 μL/mL, 6.25 μL/mL, 1.5 μL/mL and 3.125 μL/mL, respectively) [52]. More recently, in 2015 other research groups investigated the antibacterial activity of cinnamon essential oil and extracts and found similar results [53,54]. In this context, another investigation, which is particularly worthy of note, is that from Kim *et al.* They reported that cinnamon bark oil, cinnamaldehyde and eugenol at 0.01% (*v*/*v*) significantly decreased biofilm formation of enterohemorrhagic *E. coli* O157:H7 (EHEC). Another investigation focusing on Gram-negative bacteria was published by Seukep *et al.* (2013) [55] who studied the *in vitro* antibacterial activity of several Cameroonian dietary plants, including *C. zeylanicum*, against multidrug resistant (MDR) Gram-negative bacteria overexpressing active efflux pumps, which make bacteria resistant to antibiotic treatment. The tested bacteria included both reference (from the American Type Culture Collection, ATCC) and clinical strains of *E. coli*, *Enterobacter aerogenes*, *Providencia stuartii*, *P. aeruginosa*, *Klebsiella pneumoniae*, and *Enterobacter cloacae*. The bacterial efflux pumps can be blocked by various inhibitors, which restore the intracellular concentration and activity of the antibiotics. In this research the antibacterial activity was evaluated using the liquid microdilution method. Chloramphenicol was used as an antibiotic in the absence and the presence of phenylalanine arginyl ß-naphthylamide (PAßN), a known efflux pump inhibitor, which was used to show the role of efflux pumps in the resistance of the studied bacterial strains. The results revealed that the methanolic bark extract of cinnamon is able to inhibit bacterial growth, with different MIC values, ranging from 64 to 1024 μg/mL, depending on the strains. The authors concluded that the antibacterial activity of that cinnamon methanolic bark extract could be used in the treatment of infectious diseases induced by bacteria expressing MDR phenotypes [56].

Some studies showed that cinnamon extracts and essential oils could be active against oral cavity infections. Chaudhari *et al.* in 2012 [57], showed that cinnamon essential oil was active against *Streptococcus mutans* and concluded that the use of cinnamon essential oils can be a good alternative to other antibacterial compounds against the bacteria responsible for oral infections. More recently, the antibacterial activity of *C. zeylanicum* fresh leaf extract was studied against *Enterococcus faecalis*, one of the main causative factors of pulp and periapical diseases of the oral cavity. *E. faecalis* was grown both on cellulose nitrate membrane and on a tooth model system. The antibacterial activity was determined by the agar diffusion test and microdilution method. The results showed that the obtained inhibition zones vary with increasing concentration (5% to 20%) of cinnamon fresh leaf extract. Moreover, a complete inhibition of bacterial growth was registered after 12 h of contact, using NaOCl as a reference, which suggests that the cinnamon extract is active against both planktonic and biofilm forms; this was also observed *in vivo* [58]. Another piece of recent research has demonstrated that the essential oil obtained from the fresh leaves of *C. zeylanicum* is active against *S. mutans* and *Lactobacillus acidophilus* which are involved in dental plaque formation and caries development. The MIC values obtained from *S. mutans* with the tube dilution bioassay were lower than that of gentamycin (0.31 µL/mL and 0.83 µL/mL, respectively). *L. acidophilus* was less sensitive to this essential oil (1.46 µL/mL). The authors concluded that promising *in vitro* data would require *in vivo* studies to determine the dose to be used in products for oral hygiene, which have no cytotoxicity [59].

The aqueous, hydro-alcoholic and alcoholic dried inner bark extracts of cinnamon obtained using Soxhlet extraction were tested against two acne causing bacteria, *i.e.*, *Propionibacterium acnes* and *Staphylococcus epidermidis*, using the well diffusion method. The results showed that at a concentration of 5 mg/mL the inhibition zone for aqueous and ethanolic dried inner bark extracts against *P. acnes* was 18 ± 1.02 mm and 18 ± 1.6 mm, respectively. The hydro-ethanolic dried inner bark extracts were found to be inactive. The *S. epidermidis* strains were more sensitive towards these extracts, with higher inhibition zones (22 ± 1.7 mm, 22 ± 1.2 mm and 15 ± 1.8 mm for aqueous, hydro-alcoholic and ethanolic extracts, respectively). The authors ascribed the antibacterial activity to the presence of phenolic compounds such as cinnamaldehyde and eugenol, and concluded that these cinnamon extracts could be used to develop new formulations for acne treatment [31].

The antibacterial activity of cinnamon bark essential oil was also tested against 50 clinical strains of *Mycoplasma hominis* isolated from the cervical swabs of randomly selected women. *M. hominis* is responsible for bacterial vaginosis, pelvic inflammation, and pyelonephritis. The essential oil, whose main constituents was cinnamaldehyde (97% *w*/*w*), showed antibacterial and bactericidal activity, with MIC values ranging from 250 to 1000 µg/mL [60]. Another bacterium involved in sexually transmitted infection is represented by *Haemophilus ducreyi*, a Gram-negative coccobacillus, which is a strict human pathogen responsible for the development of chancroid. Due to the fact that starting from the 1970s, some strains of *H. ducreyi* have shown resistance to penicillin and its derivatives and then to sulfonamides, aminoglycosides, tetracyclines, and chloramphenicol it is of particular concern the search of new compounds given the connection between Human Immunodeficiency Virus 1 (HIV-1) and chancroid. The authors showed the antibacterial activity, expressed as MIC and minimum lethal concentrations, of the essential oil obtained from *C. verum* [61].

### 7.2. Examples of Cinnamon Applications in Food and Cosmetic Industries

Food and cosmetic products can be vectors for many harmful microbial agents that can cause infections. Foodborne pathogens are responsible for infectious diseases that are a growing public health problem worldwide, affecting about 2 million children every year, especially in developing countries. Nevertheless, foodborne diseases are not limited to developing countries, and the research on preservatives able to inhibit bacterial degradation of food and cosmetics is important for the ongoing maintenance and improvement of public health. As reported above, in recent years many investigations have shown the antimicrobial activity of cinnamon essential oil against food poisoning bacteria *in vitro*. Other investigations have studied the protective effects of cinnamon in food matrices, cosmetic products and active packaging and their ability to inhibit pathogen growth without introducing chemical preservatives that consumers could find undesirable. For instance, a recent investigation showed that the essential oil obtained from the bark of *C. cassia* can control the growth of the spoilage microorganism *L. monocytogenes* in meat products contaminated at a concentration of 5 ppm, which did not change the sensorial properties of the products. In particular, cinnamon essential oil reduces the bacterial growth rate significantly in artificially contaminated samples when compared with an untreated control [62]. Similar investigations were performed a few years back by several research groups that studied the antibacterial activity of cinnamon against foodborne pathogens, especially in contaminated meat, such as *Salmonella typhimurium*, *S. aureus* and *E. coli*, *Arcobacter butzeiri* and *Arcobacter skirrowii* [63,64,65]. The following paper is particularly noteworthy because the extract obtained from a cinnamon stick resulted to be active at room temperature (~23 °C) against *L. monocytogenes*, *S. aureus*, and *Salmonella enterica* in a food matrix different from meat and represented by cheese, suggesting that the extract is a potential natural food preservative [66]. Another interesting investigation reports the antibacterial activity of cinnamon bark essential oil and its main constituents, *trans*-cinnamaldehyde and eugenol against *Cronobacter sakazakii* and *C. malonaticus*, which are opportunistic pathogens that cause infection in children and immunocompromised adults. These bacteria are present in many food products; therefore, decreasing the bacterial count would be desirable. The antibacterial activity was assayed in liquid and vapor phases to test the strain susceptibility to both nonvolatile and volatile compounds. The results showed that the MIC values of cinnamon essential oil (ranging from 0.25 to 0.5 mg/mL) in liquid and vapor phase are similar to those registered in the same conditions for *t*-cinnamaldehyde (ranging from 0.128 to 0.3 mg/mL). Eugenol showed higher MIC values (ranging from 0.512 to 1.0 mg/mL), suggesting lower antibacterial activity. Based on these results, the authors concluded that cinnamon essential oil could be incorporated into food packaging materials or used to create an active modified atmosphere to reduce the contamination of *Cronobacter* species [67]. Another study showed that commercial essential oils obtained from the two most common species of cinnamon, *C. cassia* (leaf-branch) and *C. verum* (bark), were tested against *L. monocytogenes* NCTC 11994, *L. monocytogenes* S0580 (isolated from pork meat), *S. typhimurium* ATCC 14028, *S. typhimurium* S0584 (isolated from pig carcass), *E. coli* O157:H7 ATCC 35150 and *E. coli* O157:H7 S0575 (isolated from minced beef), *Brochothrix thermosphacta* ATCC 11509, and *P. fluorescens* ATCC 13525. The antibacterial activity was evaluated using the disk-diffusion methodand both MIC and MBC values were calculated. The essential oils showed high antimicrobial activity against the tested bacteria with MIC values lower than 1 µL/mL. The authors attributed this activity to the main bioactive constituents, especially cinnamaldehyde. They suggested that these essential oils and their main active components could be used as natural alternatives for food preservation to retard or inhibit the bacterial growth of pathogenic and spoilage bacteria and to extend the shelf life of the food products [68].

As far as the cosmetic field is concerned, Herman *et al.* (2013) showed that commercial cinnamon essential oil in a cosmetic emulsion at 2.5% concentration possesses very good antibacterial activity against several contaminants such as *P. aeruginosa* ATCC 27853, *E. coli* ATCC 25922, and *S. aureus* ATCC 29213. The antibacterial activity, evaluated with the disk-diffusion test, was found to be higher than that registered for methylparaben, used as positive control. The diameters of inhibition zones ranged from 24 to 44 mm for the cinnamon essential oil, and from 9 to 8 mm for methylparaben [5].

Another practical application for the antibacterial activity of cinnamon essential oil was reported by Hill *et al.* [69] who tested cinnamon bark extract entrapped in nanoparticles prepared with poly dl-lactide-co-glycolide (PLGA), a biocompatible polymer widely used in the pharmaceutical industry and which could be used in the food industry to deliver antimicrobial compounds to food matrices. The authors tested the antibacterial activity of the nanoparticles loaded with cinnamon extract against *L. monocytogenes* and *S. typhimurium*. The results showed that the nanoparticles exerted antibacterial activity against the tested bacteria. Therefore nanoencapsulation could be a good method to deliver entrapped antibacterial substances to pathogens in food products without a heavy influence on sensorial properties.

Table 2 forms a summary of the antibacterial studies reported in Section 7.

**Table 2:** List of cinnamon essential oils or extracts active against Gram-negative and Gram-positive bacteria.

Type of Sample	Bacteria	References
**BARK extracts, obtained with different organic solvents (ethyl acetate, acetone and methanol)**	*Klebsiella pneumonia* 13883	[45]
	*Bacillus megaterium* NRS	
	*Pseudomonas aeroginosa* ATCC 27859	
	*Staphylococcus aureus* 6538 P	
	*Escherichia coli* ATCC 8739	
	*Enterobacter cloaca* ATCC 13047	
	*Corynebacterium xerosis* UC 9165	
	*Streptococcus faecalis* DC 74	
**STEM BARK Ethanolic extract**	*Staphylococcus aureus* (MRSA)	[46]
**BARK AND CLOVE POWDER Hydroethanolic extract**	*Moraxella catarrhalis*	[47]
**Combination of piperacillin and cinnamon BARK essential oil**	*E. coli* (β-lactamase-producing)	[49]
**Essential oil obtained by hydro-distillation of cinnamon BARK**	*Salmonella typhi*	[50]
	*Salmonella paratyphi A*	
	*Escherichia coli*	
	*Staphylococcus aureus*	
	*Pseudomonas fluorescens*	
	*Bacillus licheniformis*	
**Essential oil obtained by hydro-steam distillation of cinnamon BARK**	*Escherichia coli* O157:H7	[51] [52] [53]
	*Yersinia enterocolitica* O9	
	*Proteus* spp.	
	*Klebsiella pneumonia*	
**Essential oil (BARK and fresh LEAVES)**	*Escherichia coli* O157:H7	[52] [53] [54] [56] [58] [59] [60] [61] [63]
	*Yersinia enterocolitica* O9	
	*Proteus* spp.	
	*Klebsiella pneumonia*	
	*Streptococcus mutans*	
	*Lactobacillus acidophilus (fresh leaves)*	
	*Mycoplasma hominis* (bark)	
	*Haemophilus ducreyi* (E. O from *C. verum*)	
	*L. monocytogenes (*bark*) (*E.O from *C. cassia*)	
	*Salmonella typhimurium*	
**Methanolic extract of cinnamon BARK**	*Escherichia coli*	[55]
	*Enterobacter aerogenes*	
	*Providencia stuartii*	
	*Pseudomonas aeruginosa*	
	*Klebsiella pneumoniae*	
	*Enterobacter cloacae*	
**Fresh LEAF extract**	*Escherichia coli* O157:H7	[52] [53] [57]
	*Yersinia enterocolitica* O9	
	*Proteus* spp.	
	*Klebsiella pneumonia*	
	*Enterococcus faecalis*	
**Aqueous, hydroalcoholic and alcoholic dried inner BARK extracts (Soxhlet)**	*Propionibacterium acnes* (hydroethanolic extracts inactive)	[30]
	*Staphylococcus epidermidis*	
**Cinnamon BARK extracts**	*Salmonella typhimurium*	[62]
	*S. aureus*	
	*E. coli*	
**Extract obtained from cinnamon STICK**	*L. monocytogenes*	[65]
	*S. aureus*	
	*Salmonella enterica*	
**BARK essential oil (tested in liquid and vapor phases)**	*Cronobacter sakazakii*	[66]
	*C. malonaticus*	
**Commercial essential oils from *C. cassia* (LEAF-BRANCH) and *C. verum* (BARK)**	*L. monocytogenes* NCTC 11994	[67] [5] [64]
	*L. monocytogenes* S0580	
	*S. typhimurium* ATCC 14028	
	*S. typhimurium*	
	*E. coli* O157:H7 ATCC 35150	
	*E. coli* O157:H7 S0575	
	*Brochothrix thermosphacta* ATCC 11509	
	*P. fluorescens* ATCC 13525	
	*P. aeruginosa* ATCC 27853	
	*E. coli* ATCC 25922	
	*S. aureus* ATCC 29213	
	*Arcobacter butzeiri*	
	*Arcobacter skirrowii*	
**Nanoparticles loaded with cinnamon BARK extract**	*L. monocytogenes*	[68]
	S. typhimurium	

## 8. Toxicological Aspects

Despite the above reported studies that promote the use of cinnamon applications in food and cosmetic products, the oral ingestion or skin applications of cinnamon or its components (*i.e.*, cinnamaldehyde, eugenol, and cinnamic acid) is not always advisable and is recommended only in very small doses. Cinnamon oil should be diluted to less than 2% before oral use [70]. Moreover, it is recommendable to avoid the ingestion of cinnamon bark oil *per os* to patients suffering from liver conditions, in case of alcoholism and when taking paracetamol. This recommendation is related to the capacity of cinnamaldehyde to deplete glutathione [71]. Regarding cinnamon bark, it appears to be safe for most people when taken by mouth in amounts up to six grams daily for six weeks or less [70]. We must also point out that no drug interactions are reported for *C. zeylanicum.* Differently, *C. cassia* bark (at the dose of 2 g in 100 mL) retarded the *in vitro* dissolution of tetracycline. HCl. In fact, only 20% dissolved within 30 min in contrast to 97% when only water was used. Due to the fact that this dose is not uncommon, it is recommended not to use tetracyclines together with cinnamon [72]. Finally, the ingestion of cinnamon oil may cause central nervous system depression, predisposing the patient to aspiration pneumonia [21].

As far as topical cinnamon applications are concerned, it should be remembered that cinnamon is used as a constituent of personal hygiene products, as toilet soaps, mouthwash, toothpaste, as ingredients of beverages and baking products and as flavoring of gums. Therefore, contact dermatitis, perioral dermatitis, stomatitis, gingivitis, glossitis, sub-mucosal inflammation and alteration of the surface epidermis should occur after the contact with products containing cinnamon. Intraoral reaction clinical manifestations consist of pain, erythema, ulcerations, fissures, vesicles, desquamation and hyperpigmentation, and white patches. The main responsible for these manifestations are considered Cinnamaldehyde and cinnamic acid are considered to be mainly responsible for these manifestations because they act as membrane irritants. The severity of the local mucosal reaction depends on the duration of contact and systemic symptoms such as nausea and abdominal pain are rare [33].

## 9. Conclusions and Recommendations

This paper has reviewed the available references regarding the antibacterial effects of cinnamon and its active constituents, published over the last five years. It has shown that the antibacterial activity of cinnamon is due to bioactive phytochemicals such as cinnamaldehyde and eugenol. Cinnamon use in food products and cosmetics could be a good strategy to reduce or avoid bacterial degradation and thus to reduce the incidence of infection caused by food and cosmetics. In fact, cinnamon is not harmful when used in correct conditions. Regardless, long standing excessive use is not recommended.

Moreover, cinnamon could be used to treat infectious disease. However, there is a lack of clinical trials on the antibacterial effects of cinnamon and its chemical constituents, and therefore its clinical efficacy is not clear. In addition, it is well known that cinnamon essential oil toxicity is one of the most important problems, as some essential oils show the above reported adverse effects on human cells, such as cytotoxicity and cell death. Therefore, the application of natural products in the treatment of infectious diseases may be considered an interesting alternative to common antibiotics, possessing different side effects. In addition, cinnamon can be suggested as an alternative to synthetic antibiotics, especially for the treatment of antibiotic-resistant bacterial infections.

Furthermore, we provide a brief summary of the history, traditional uses, phytochemistry and clinical impacts of cinnamon to provide a better view of this spice and herbal medicine. Finally, we recommend that further studies should be performed on the toxicity of cinnamon prior to its clinical use; studies on the mechanism of the antibacterial effects of its extracts and essential oils; on the separation, purification and identification of the most effective antibacterial constituents of cinnamon and their food- and drug-interactions; and clinical studies to examine the antibacterial effects of extracts and essential oils of cinnamon and its bioactive constituents in different infectious diseases.
